# Exploring vaccination attitudes in African communities in Canada: A mixed-methods study protocol

**DOI:** 10.1371/journal.pone.0319584

**Published:** 2025-04-28

**Authors:** Obidimma Ezezika, Zantae Pellitier, Sonia Muhimpundu, Christian Daboud, Meron Mengistu, Omolola Olorunbiyi, Christian Hines, Mekoya Wondrad, Joanne Kearon, Reforce Okwei, Kingsley Anukam, Dominic Alaazi, Godwin Arku

**Affiliations:** 1 Global Health & Innovation Lab, Faculty of Health Sciences, Western University, London, Ontario, Canada; 2 School of Health Studies, Western University, London, Ontario, Canada; 3 London InterCommunity Health Centre, London, Ontario, Canada; 4 Middlesex-London Health Unit, London, Ontario, Canada; 5 Ethiopian Association London, Ontario, Canada; 6 Ghana Association of London and Middlesex, London, Ontario, Canada; 7 Igbo Association of London and Area, London, Ontario, Canada; 8 Faculty of Geography and Environment, Western University, London, Ontario, Canada; PLOS: Public Library of Science, UNITED KINGDOM OF GREAT BRITAIN AND NORTHERN IRELAND

## Abstract

**Introduction:**

Vaccine hesitancy is a complex issue influenced by many interacting factors. While literature on its contributing causes continues to expand, there is limited research on the contextual and cultural dynamics that shape vaccine hesitancy among African-born individuals in Canada. Identifying and understanding these factors is critical in developing targeted health interventions that address specific barriers to vaccination within this community. The study aims to explore the unique socio-cultural and context-specific elements of vaccine hesitancy among African community members living in Canada.

**Methods and analysis:**

The study will use a mixed-methods approach to investigate vaccine hesitancy among African community members living in Southwestern Ontario. In the qualitative study, we will conduct semi-structured interviews and participatory focus groups within each of the selected study areas: London, Windsor and Chatham-Kent. The qualitative data will be collected, transcribed and then analyzed thematically using NVivo 12. For the quantitative study, we will provide participants with surveys to accurately assess the predictors of vaccine hesitancy. The quantitative data will be analyzed using logistic regression to explore how socio-cultural influences, trust, and accessible information impact vaccine hesitancy.

**Discussion:**

This study addresses a significant gap in existing literature by providing cultural and contextual insights on the drivers of vaccine hesitancy among African-born individuals. Using a mix-method design, the study offers a rich understanding of the influences shaping vaccine decision-making. The findings will support the development of health policies and interventions aimed at improving overall health outcomes for African communities within Canada.

## Strengths and limitations of this study

- The qualitative and quantitative data collected will advance literature on understanding the cultural and contextual factors of vaccine hesitancy among African-born individuals living in Canada.
- A limitation of the study is its generalizability due to its focus on African-born individuals living in Southwestern Ontario (SWO). Therefore, conclusions drawn may not fully represent the experiences and attitudes of African community members living throughout Canada.

## Introduction

Vaccination is widely recognized as a key public health intervention [1–3]. The World Health Organization estimates that immunization programs have contributed to saving 154 million lives globally [4], helping to lower overall disease burden [3,5]. Despite these efforts, vaccine uptake remains a challenge due to differing perceptions regarding the safety and efficacy of vaccines [6]. This growing skepticism, defined as “a delay in acceptance or refusal of a vaccines service despite the availability of services” [7], has emerged as one of the top ten global health threats [8], highlighting a critical challenge to ongoing public health efforts. A study published in 2018 further highlights this, as it found that Black healthcare workers, including African-born individuals, were least likely to receive an influenza vaccine between 2011–2013 compared to participants of different racial groups [9].

This study explores vaccine hesitancy among African community members who arrived in Canada, focusing on Southwestern Ontario. Research on COVID-19 vaccine hesitancy in Black populations in Canada, including African community members, has revealed key barriers such as distrust in governments, negative perceptions of vaccine safety and concerns regarding coercion to receive a vaccine [10–13]. However, vaccine hesitancy is not limited to the COVID-19 vaccine; it affects other vaccines such as Human Papillomavirus (HPV) vaccine [14–16].

Among African-born individuals living in Canada, there is a need for a more nuanced understanding of the drivers of vaccine hesitancy and how they develop. This project aims to generate a deeper and more comprehensive understanding of the factors of vaccine hesitancy using a participatory action research approach that directly engages African community members in Southwestern Ontario. By prioritizing their lived experiences and diverse perspectives surrounding vaccines, this study will explore (1) the underlying factors contributing to vaccine hesitancy, examining how these factors intersect across sub-groups within the African communities in Canada, and (2) uncover contextual settings where vaccine hesitancy emerges, examining the unique knowledge and decision-making processes that shape attitudes towards vaccines among African community members who arrived in Canada.

Through ongoing engagement with key partners, community members and leaders, this study aims to provide data on the barriers and facilitators of vaccine uptake within this demographic group. By centering participants’ voices, this approach will identify and quantify important factors associated with vaccine hesitancy, ultimately providing valuable insights for Canadian public health officials and policy makers to create more tailored and culturally relevant health strategies and policies.

## Methods and analysis

### Qualitative study design

The qualitative study will involve one-on-one semi-structured interviews with African community members residing in Southwestern Ontario, specifically including urban areas such as London and Windsor, alongside the rural region of Chatham-Kent. 60 participants will be recruited to participate in semi-structured interviews with equal numbers represented across the three regions (i.e., 20 interviews per city). The interview guide (Table 1) was developed following consultations with the Community Steering Committee (CSC), a participatory body involving community members, partners and experts. During the consultation meetings, the CSC provided guidance and oversight to ensure that the interview guide aligns with the needs and values of African community members living in Southwestern Ontario, while achieving the stated aims of the study.

**Table 1:** Interview guide.

Socio-demographic questions
1.How many years have you lived in Canada?
2.What is your highest level of education?
3.What is your average level of income?
4.What is your primary (main) language?
5.What is your age?
6.What is your country of origin (nationality)?
7.Do you have children? If yes, how many?
8.Do you have any children under the age of 5 years old?
9.What is your religion/faith?
10.What is your sex?
**Open-ended questions**
11.What is your understanding of vaccines and their role?
12.Would you accept vaccines for yourself?
a.PROMPT: What are the reasons you get vaccinated?
13.If you want to get vaccinated, what helps you to get vaccinated?
14.If you want to get vaccinated, what are the challenges of getting vaccinated?
a.POMPT: What might stop you from getting vaccinated)?
15.In your opinion, what reduces vaccine confidence?
16.Do you feel pressured to get vaccinated?
a.What are the reasons you feel pressured (e.g., for work, school, family, or other reasons?)
17.Where do you get your vaccine information from?
18.Do you trust the source(s) that you get your vaccine information from?
19.How does your community influence vaccine information and trust?
20. Has your child ever been vaccinated?
21.What is your experience vaccinating your child?
22.Would you accept recommended vaccines for your child? Can you explain why or why not?
23.Some vaccines are required. If you had a choice, would you vaccinate your child? Can you explain why or why not?
a.PROMPT: Would you vaccinate your child before the age of 5 years?
b.PROMPT: Would you vaccinate your child before the age of 5 years?
24.Are there certain vaccines you trust more or less than others? Why?
a.PROMPT: Can you tell me about your feelings toward different vaccines, such as those for measles, polio, HPV, and COVID-19?

Following the semi-structured interviews, participants will be invited to participate in focus group discussions (FGDs) based on the results of the semi-structured interviews.

### Quantitative study design

The quantitative study of the project will use a cross-sectional survey to assess the different variables that might influence vaccine hesitancy among African-born individuals living in the selected SWO cities. Using three validated survey tools - *Centre for Disease Control Vaccine Confidence Survey* [17], *Religious affiliation and philosophical and moral beliefs about vaccines* [18], and *The Parent Vaccine Hesitancy Survey tool* [19], survey items have been adapted and combined into a single survey tool (see Appendix 1).

### Participant and public involvement

Before starting the qualitative data collection process, which involves semi-structured interviews and focus discussion groups, and the quantitative data collection process which involves the survey tool (see appendix 1), the project team held group consultations with community and health representatives in London, Ontario. A total of 41 partners were identified through web search of African community organizations, settlement services, and local government sites (e.g., City of London), as well as recommendations from the project team’s networks. The focus of the search was for individuals representing organizations that have an interest or involvement in the health of African-born individuals living in SWO. For the first group consultation, 36 partners representing several organizations were contacted and invited, including the African Canadian Federation of London and Middlesex Area, African Community Council, London & Middlesex Local Immigration Partnership, Middlesex-London Health Unit, London Cross-Cultural Learner Centre, Ethiopian Community Association London, Nigerian Association London and Area, Igbo Association Of London and Area, Ghanaian Association of London-Middlesex, Rwanda Canadian Community London, Sudanese Canadian Community Association London Ontario and Area, Eritrean Canadian Association of London & Suburbs, United Canadian Liberian Association of London, City of London, and London InterCommunity Health Centre.

On August 10, 2024, the first meeting at the University of Western Ontario gathered eight individuals from the Sudanese Canadian Association, Cross Cultural Learners’ Centre, Ghana Association of London and Middlesex, Igbo Association of London and Area, Ethiopian Community Association London, Middlesex-London Health Unit, and London InterCommunity Health Centre. The group reviewed the project’s aim, design, population of interest, data collection methods, and interview guide. 12 key suggestions emerged and can be found in Table 1. Key recommendations for refining the study included broadening eligibility to all timeframes in Canada, implementing purposive sampling by residency duration, adjusting questions to capture detailed vaccine experiences, and addressing key facilitators and barriers. Suggestions also included using accessible language, incorporating community and religious perspectives, building an environment of trust, ensuring study independence from government, involving community leaders in recruitment, and gathering socio-demographic data and other key predictors of vaccine hesitancy.

Following the initial meeting, the proposal was revised to incorporate the suggested changes. Table 1 outlines the action steps taken. The updated proposal was shared with participants from the first consultation session.

A second meeting was held on October 19, 2024, and invited the individuals who attended the first meeting. In this meeting, the project proposal was shared for the group to provide any further feedback. Table 2 provides the eight suggestions that emerged from the second meeting. Recommendations included ensuring a diverse sample of African communities using purposive sampling across socio-demographic variables, enlisting community leaders for recruitment, and considering a community facilitator. Neutral language in the interview guide was advised by the CSC to avoid any bias or influence over the participants responses. Terms like “vaccine hesitancy” and “African immigrant” were suggested for rephrasing. In the interview guide, the term “vaccine hesitancy” was suggested to be reworded to “vaccine confidence” or “perceptions towards vaccines” to prevent framing the discussion around vaccines in a negative light. The CSC also discussed replacing the term “African immigrant” with “African community members who arrived in Canada” or “Individuals born in Africa” to ensure inclusivity in the terminology used. Political views were added to the socio-demographic questions, and supplementary vaccine information, such as schedules and vaccine requirements, will be made available to participants on request.

**Table 2:** List of suggestions by the Community Steering Committee at the first consultation meeting (August 10, 2024), and changes.

Suggestions from consultation meeting		Action plans
**Participation Criteria**		
1	Expand the inclusion criteria to include newcomers, as well as those who have been living in Canada for some time.	The original eligibility criteria included African immigrants living in Canada for fewer than six years. The participant inclusion criteria now include individuals who have immigrated to Canada from an African country, regardless of when they arrived.
2	Use purposive sampling to ensure all individuals are represented in the study and have clear definitions of the participant categories.	Participants will be selected based on their time spent in Canada (less than 1 year, 1–3 years, 4–9 years, and 10 or more years); their African region of origin (Northern, Southern, Eastern, Western); whether they have children (children under five, pre-adolescent/adolescent children, those with adult children, no children); and their religious/spiritual affiliation (Christian, Muslim, Jewish, other).
**Interview Guide/Data Collection**		
3	Encourage participants to provide specific cases or stories of vaccines and personalize questions instead of asking general ones	Questions were framed in first-person language to encourage participants to draw on their personal experiences and opinions, as well as to encourage elaboration with prompts such as *why or why not* and *please explain.* In addition, questions were personalized by using first-person language, incorporating terms like “you” instead of posing them in a more general or impersonal manner.
4	Explore the barriers and facilitators to vaccination, identify key health information sources, and assess the influence of having children on vaccine hesitancy.	The interview guide was revised to incorporate questions addressing the recommended areas, including barriers, facilitators, health information sources, and childhood vaccines.
5	Consider the potential difference in vaccine hesitancy among adults with children.	The proposal integrates this suggestion in two ways. The first is in the sampling criteria. The sample will include participants with and without children, focusing on those with children under five, pre-adolescent/adolescent children, those with adult children and no children. The second is in the interview guide questions.
6	Collect social determinants of health data.	The interview guide and survey questions encompass social determinants of health.
7	Build an environment of trust in focus group discussions.	The facilitation of focus group discussions will begin with a preamble highlighting essential aspects of engagement and participation, including a set of ground rules focused on mutual respect, the use of respectful language, and fostering a healthy discourse. It will be clarified that the study has no affiliation with any government bodies and that the research goal is not to promote specific viewpoints but to offer participants a platform to share their voices on the issue. While women-only focus groups were suggested as an option, they have not been applied in this study.
**Language Accessibility**		
8	Make language more accessible by using less jargon and offer study contents in other languages of preference.	The language during recruitment and data collection will not include jargon and will use simple terms. In addition, the inclusion criteria now include participants who speak French (original plan only included English), with interviews to be offered in in French if requested.
**Recruitment Strategy**		
9	Involve community and religious leaders as a way of getting their perspective about vaccine hesitancy in their communities. Community members look up to these leaders so it’s important to receive their insights.	Community and religious leaders will be invited to participate in the study to ensure their experiences are represented.
10	Make it clear that the study/researchers are not connected to a governmental body.	Throughout outreach, recruitment, and the data collection process, participants will be informed that the study operates independently of any government body and has no conflicts of interest. This will be clearly communicated in the recruitment materials (e.g., recruitment emails, telephone scripts, and Letters of Information/Consent), as well as in the preamble statements for interviews and focus groups.
11	Engage African community leaders and associations to identify and recruit interested participants through their memberships.	Contact information such as email addresses and phone numbers stored in community-based associations will be leveraged upon their provision of and consent to reach out to eligible individuals on our behalf letting them know if they are interested in contacting the research team.
**Research Design**		
12	Consider providing potential predictors of hesitancy.	A quantitative study has been included in the project to complement the qualitative study, supporting the analysis of associations between variables.

**Table 3:** List of suggestions by the Community Steering Committee at the second consultation meeting (October 19, 2024), and changes.

Suggestions from consultation meeting		Changes
**Participation Criteria**		
1	Include a sample representing diverse African communities.	The sampling strategy will be purposive, recruiting individuals across four variables: African region, years in Canada, those with and without children, and religion/faith.
**Recruitment Strategy**		
2	Build trust with community leaders and informal leaders first to support the recruitment.	Community and informal leaders are included in the outreach and recruitment plan to support the identification and connection of community members to the research project team.
3	Hire a community leader to facilitate the focus group discussions.	Hiring a community facilitator has not been applied to this study as this would have required additional resources. This includes additional funding for hiring and training to support facilitation.
**Interview Guide/Data Collection**		
4	Include a question regarding political views or political affiliation for the socio-demographic questions.	A question asking participants what their political affiliation is has been included in the interview guide and the survey.
5	During recruitment and data phases, the study will reinforce the importance of providing participants with a platform to share their views on vaccines, ensuring that their voices are heard and valued.	Recruitment documents and interview preambles will use language and key terms to emphasize the value of different perspectives and voices, free of expectations from the research team or results.
6	Frame the language of the interview guide as neutral.	Interview guide questions were re-framed in two ways. First, questions that implied existing hesitancy and challenges or existing confidence and drivers were re-framed to be neutral and open-ended. Second, questions that used the word ‘hesitancy’ were changed to ‘perception’ or ‘confidence’.
**Reframing the Study’s Language**		
7	To ensure that the study does not influence that participants’ responses, the language used in the study will be modified to be neutral. The terms ‘hesitancy’ and ‘African immigrant’ will be reworded based on CSC’s recommendations, to use more neutral and inclusive terminology.	‘Vaccine hesitancy’ will be replaced with ‘vaccine perception’ and ‘vaccine confidence’ in the study. In addition, ‘African immigrant’ will be replaced with language such as ‘African community members who arrived in Canada’ and ‘Individuals born in Africa’.
**Supplementary Documents**		
8	Prepare for questions about vaccine informational sources to provide to participants if requested.	Vaccine information will be provided to participants in two ways. First, the interview preamble will briefly list mandated and voluntary vaccines to support responses of some questions. Second, a document listing sources that outline mandated and voluntary vaccines will be shared with participants who request them.

### Participant selection criteria

To ensure a diverse representation centering the experiences of African community members as it relates to vaccine hesitancy, the team will identify potential participants based on key variables, including years of settlement in Canada, the African region from which they emigrated, number of children and their age, and their religious/spiritual affiliations. Participants will be selected based on their time spent in Canada, categorized into four groups: less than 1 year, 1–3 years, 4–9 years, and 10 or more years, to observe how vaccine hesitancy evolves over time. They will also represent the four major African regions—East, West, North, and Southern Africa—to capture cultural diversity and potential regional differences in vaccine attitudes. The sample will include participants with and without children, focusing on those with children under five, pre-adolescent/adolescent children, and those with adult children or no children. Religious diversity will also be considered, with participants from Christian, Muslim, Jewish, and other spiritual affiliations.

To be enrolled in the qualitative and quantitative study, eligible participants will include individuals who have immigrated to Canada from an African country, regardless of when they arrived and of their status. This broad inclusion captures individuals who resided in Canada for a few months as well as those residing in Cananda for over a decade. Moreover, the study will encompass documented and undocumented African born individuals, as well as those who entered Canada on temporary resident visas (i.e., study permits, work permits) or are currently seeking asylum. All participants must be at least 18 years old and proficient in English or French. Additionally, eligible participants must reside in one of the selected cities in Southwestern Ontario: London, Windsor and Chatham-Kent.

The qualitative and quantitative study will exclude individuals who were not born and immigrated to Canada from a country in Africa, including individuals born in Canada or anywhere outside the African region. Additionally, individuals under the age of 18 will be excluded from participating in the study. Finally, those who are unable to speak English or French and do not reside in one of the selected Southwestern Ontario cities – London, Windsor or Chatham-Kent - at the time of study will also be excluded.

### Sampling

The sampling strategy used in the qualitative component of the project will be purposive, ensuring that the study captures a wide range of experiences and perspectives relevant to our research questions, which include socio-demographic considerations and cultural contexts. Recruitment of participants eligible for the semi-structured interviews will be completed by May 2025. If recruitment challenges occur, adjustments may be made to ensure appropriate representation across the four sampling variables: years of settlement in Canada, African region from which they emigrated, number of children and their age, and their religious/spiritual affiliation. Interviews will be conducted in each city. Additionally, FGDs will be conducted in the three cities (i.e., 1 FGD in each city). Each FGD will consist of about ten (10) members (lasting approximately 90 mins long). Participant recruitment for the FGDs will be completed by August 2025.

The quantitative study will use convenience sampling and snowball sampling to recruit 374 participants for the survey in the study areas. To calculate the sample sizes needed to generate representative data of African community members living in Southwestern Ontario, relevant population data was collected using each selected city’s published census [20]. It was determined that the population of African community members residing in London totals (8010), Windsor (5055) and Chatham-Kent (270). To calculate the sample size needed for the vaccine perception project (VPP) across the study areas (London, Windsor and Chatham-Kent), the population of each selected area was calculated relative to the combined total population. This included London with 8,010 individuals, Windsor with 5,055 individuals and Chatham-Kent with 270 individuals, resulting in a combined total of 13,355. Using this number, the data was then entered into Qualtrics to establish a necessary sample size, which was determined to be 374 participants. From this target, the proportional representation for each study area was calculated: London will contribute 224 participants, Windsor will account for 142 participants and Chatham-Kent will provide 8 participants. The proportional sampling from each city ensures adequate representation based on its relative population size. Participant recruitment for the quantitative component is expected to be completed by August 2025.

With the established sampling strategy for both qualitative and quantitative study components, trained research assistants and research team members will a targeted recruitment strategy to effectively reach eligible participants within the study areas. Recruitment will focus on several outreach activities and referrals from individuals participating and the CSC. Having determined our targeted participants for each selected SWO city, we propose that utilizing the following recruitment strategies will enable us to reach participant targets:

- In-Person Recruitment - Research assistants will get into contact with the ‘point-person’ in the community to assist with disseminating information about the Vaccine Hesitancy Project in churches and mosques.
- Calls/Emails - Contact information such as email addresses, and phone numbers stored in community-based associations will be leveraged upon the provision of and consent by the CSC to reach out to eligible individuals on our behalf letting them know if they are interested in contacting the research team.
- Community and social events - Research assistants can attend community, and social events first introduce the team and project and provide contact information for any eligible, interested individuals.
- Social Media Platforms - Social media platforms such as WhatsApp and Facebook (with niche groups specific to African community members who arrived in Canada) will be used to share information regarding the quantitative surveys of the project. This offers a tailored approach to increase the likelihood of reaching target participants.
- Referrals - Following participant interviews and surveys, they will be asked if they know anyone who might be interested in participating. Study participants will share the recruitment invitation with potential participants, which includes the study team’s contact information. Should others be interested in participating, they will contact the study team.

### Data collection

#### Interview guide.

Qualitative data will be collected through semi-structured interviews using the interview guide that emerged from the meeting with our CSC (Table 1.). Interview sessions will be held virtually or in-person and will last for 60 minutes. Zoom will be the platform used for online interviews, and in-person semi-structured interviews will occur at a mutually convenient location (e.g., a private room in a community center, public library, or on campus), as determined by the participant and the research team. Online interviews will have video disabled, and the interviews will be audio recorded. Data collection from the semi-structured interviews will be complete June 2025 and results will be finalized by July 2025. Additionally, for the in-person interviews, an audio recorder will be used for audio recording with permission from the participants. For interviews, arrangements will be made to transfer $15 to each participant covering an honorarium.

#### Focus group discussions.

Moreover, to supplement and ensure diversity in the qualitative data collected, there will be one focus group discussion held in London, Windsor and Chatham-Kent that will comprise of 10 community members. These focus group discussions will occur at rented community centers and will be audio-taped with the permission of participants, expecting to last for 90 minutes. The participatory based group discussions will enable participants to explore and expand on themes that emerged from the semi-structured interviews, allowing for findings to be enhanced and contextually relevant through collective insights. Moreover, the discussions will take the form of an open dialogue giving participants a safe environment to not only freely express their views but exchange ideas. The data collected from the focus group discussions is scheduled to be completed by September 2025 with results expected by October 2025.

Each session will be guided by a trained facilitator who will moderate the discussion using a list of structured questions. However, to allow spontaneity on conversations that might emerge, the facilitators for each session will remain flexible. For FGDs, arrangements will be made to transfer $20 to each participant covering an honorarium.

#### Survey.

Quantitative data will be collected using a cross-sectional survey (refer to appendix 1). The survey will be conducted online using Zoom and is expected to take approximately 30 minutes to complete. During the session, the researcher will guide the participant through each question and securely record their responses using Qualtrics.

To ensure that the survey being used in the quantitative study was culturally relevant and applicable to African community members who arrived in Canada, the research team conducted a thorough search of the literature to find existing validated surveys tools, this process involved assessment of their strengths and weaknesses through consensus, with the CDC Confidence Survey Tool initially being considered as potential survey tool.

The CDC Confidence Survey Tool was developed to capture population-wide studies of vaccine hesitancy related to the COVID-19 vaccine specifically in the United States. Acknowledging the variations of health outcomes among different demographics in the United States, (specifically individuals of Black and African descent) the CDC then established a guideline for minority communities which stated that with thorough community assessment which establishes the variations in needs, concerns or problem areas of that community, the CDC Confidence Survey Tool can be useful to then determine factors i,e. (vaccine hesitancy) hindering vaccination uptake [21]. The guideline states that the initial needs assessment should be accomplished through a focus group, community gathering. Following the guideline, we have achieved that with our meeting held on August 10, which acted as a medium to gain insight in areas that are relevant to the African community in SWO and how those insights can aid in refining the survey tool.

To ensure the adapted survey tool was comprehensive, three validated tools were combined – The CDC Confidence Survey [17], The Parent Vaccine Hesitancy Survey, which was piloted in various WHO regions [19] and the Religious Affiliation and Philosophical belief about Vaccines tool, which offer proportional racial representation [18]. Initially, we attempted to locate a single validated survey that aligned with the themes that emerged from our consultation meetings. While the CDC Vaccine Confidence survey met many of the thematic criteria, it required modifications to include: a wider range of vaccines beyond COVID-19, critical topics such as religious influences and parental attitudes toward childhood vaccines, which were either missing initially or inadequately addressed. To fill these gaps, 2 supplementary questions were integrated from The Parent Vaccine Hesitancy Survey such as 1. “*Do you believe vaccines can protect children?”* and 2. *“Do you think most parents like yourself have their children aged 0-5 years vaccinated with all the recommended vaccines?”* and 1 question related to religious beliefs was included to explore the impact of religious influences on attitudes towards vaccines was integrated from the Religious and Philosophical moral beliefs survey: *“Does your religion or spiritual beliefs prohibit its members from getting vaccines”.*

To refine and streamline the survey, repetitive and irrelevant questions were removed. Duplicate questions such as “*Have you ever received an incentive to get a COVID-19 vaccine?*” that were found in both child and adult focused questions were eliminated. Questions that were outside of the study’s scope such as “*What is your zip code?*” and “*What brand of COVID-19 vaccination did you receive”* were also deleted. After these revisions, the final survey (see appendix 1) includes 72 questions: 58 originating from the CDC Vaccine Confidence Survey tool [17], 2 from the Parent Vaccine Hesitancy Survey Tool [19] and 1 from the Religious Affiliation and Philosophical belief about Vaccines tool [18].

The survey (see appendix 1) will include different questions to gather measurable, quantifiable data. These questions will consist of Likert scale questions, yes or no questions and, open-ended text box questions, each serving a distinct purpose. Likert scale questions will be used to assess levels of trust or agreement. An example of this is a question regarding trust in healthcare information “*Do you trust your healthcare provider in offering reliable vaccine information*” with response options ranging from “Not at all” to “Completely”. Yes or No questions will be used to capture more straightforward and actionable responses. For example, the survey includes the following question exploring vaccine incentives, “*Have you heard of cash prizes or other rewards being offered in your area to people who get a vaccine*?” with response options Yes, No or Maybe. Lastly, open-ended text box questions are designed to allow participants to provide specific demographic details. Questions such as “*How old are you*?” will be followed with a provided space to allow participants to enter in their exact age, allowing for more personalized data. The data collection for the surveys will be completed by October 2025 with results expected by December 2025. Additionally, arrangements will be made to transfer $10 to each survey participant covering an honorarium.

### Data analysis

The iterative plan for collecting and analyzing the qualitative data will use a thematic analysis approach, to effectively capture individual and group level insights from the semi-structured interviews and focus discussion groups respectively. First, the interviews and focus groups will be audio-recorded and transcribed verbatim. Second, transcripts will be checked and edited, as necessary, for accuracy and completeness, and to remove any identifying information. Third, interview transcripts will be analyzed utilizing NVivo 12 Plus through directed content analysis, to identify themes and patterns across the data. When analyzing the focus group discussions, an additional layer will be applied that focuses in on group dynamics. Thematic analysis in this context is enhanced by the participatory and interactive nature of the in-person focus groups, allowing for the designated team members to examine what participants say in this context and how themes emerged through the group interactions. Fourth, two team members will independently analyze the interview transcripts for factors, meeting periodically to adjudicate coding differences and create a consensus template. Fifth, as an additional reliability check for coding, a third reviewer will code a subset of interviews and resolve conflicts between the two researchers. Sixth, team members will individually review the transcripts to identify preliminary themes. Seventh, team members will collectively review the preliminary themes, resolve any mislabeling or duplication, and agree on a final list of themes and sub-themes. We will rely on the Consolidated Framework for Implementation Research (Damschroder et al., 2022) to identify barriers to and affordances in vaccine hesitancy. We have used this approach for other vaccine programs around the HPV vaccine in Rwanda [22] and the meningitis vaccine in Burkina Faso [23].

The quantitative data collected from the surveys will be analyzed using regression modeling. There is considerable literature that explores the relationship between various variables of vaccine hesitancy using regression models. Piers (2022) conducted a systematic review that determined studies exploring vaccine hesitancy of the COVID-19 vaccine used regression modeling to provide precise and accurate predictors of vaccine hesitancy [24]. For the nature of the quantitative study, regression modeling is best suited as it will allow us to explore the relationship between the different variables gathered from the survey and vaccine hesitancy, alongside determining the most influential causes of vaccine hesitancy. Based on the questions format included in the survey we can expect to use Logistic (binary) regression: To assess binary outcomes in relation to vaccine hesitancy using the predictor variable (e.g., Trust - Do you trust your healthcare providers in offering reliable vaccine-related information?) against the odds outcomes (vaccine hesitancy).

### Ethics and dissemination

This study has been approved by the Health Sciences Research Ethics Board (HSREB) at the University of Western Ontario (Project ID: 126183). Eligible participants will be informed about the study’s purpose and their right to withdraw at any time without any consequences. Recruitment will occur over the phone, email and in-person with each participant receiving a Letter of Information and Consent (LOI/C) stating the study’s purpose, participants roles, time commitments, confidentiality, audio-recording, honorarium and withdrawal procedure.

For participants receiving a recruitment email, the Letter of Information and Consent (LOI/C) form will be attached to the email containing the consent form link. To participate in the study, recruited participants must read, e-sign, and submit the consent form via Western Qualtrics before any research-related activities begin. Participants may be recruited via telephone if the research team is contacted by interested participants via telephone. If participants express further interest, then an email will be sent containing the LOI/C. CSC community representatives will contact their associations’ members and key religious and community leaders to share the recruitment email/materials with them. The email will contain the email address and phone number of the Principal Investigator and Research Coordinator to reach out to if they wish to participate. Interested individuals will have to contact the PI/Research Coordinator if they would like to participate or have further questions.

Once individuals have expressed their interest, they must submit their e-consent form (via Qualtrics) to the research team before research activities begin. The LOI/C forms will outline participants’ roles, time commitment, the necessity of audio recording, confidentiality, potential risks, honoraria, FGD consent processes, survey processes, and the procedure for discontinuing or withdrawing from the study. To note, a separate LOI/C form will be created and distributed for each activity (interviews, focus groups, and surveys).

## Discussion

Understanding and identifying drivers that contribute to vaccine hesitancy among African community members who arrived in Canada is crucial when developing culturally sensitive health interventions and health messaging. African-born individuals face unique challenges compounded by a complex interplay of systemic, structural and social determinants of health. A study published in Manitoba on primary healthcare found that among 83 families originating from 15 African countries, revealed that there were significant barriers when accessing healthcare and the importance of integrating culturally applicable health programs [25]. Similar trends were also seen in the area of mental health, where African-born individuals were less likely to utilize mental health services compared to their White Canadian counterparts [26], further emphasizing the importance of culturally relevant and tailored health interventions.

By gaining insights into the cultural and contextual factors that act as drivers for vaccine hesitancy, public health officials and policymakers can develop targeted public health interventions and strategies that fit with the needs and concerns of African communities in Canada, ultimately promoting healthier outcomes among this demographic.

While the proposed study contributes to the growing literature surrounding vaccine hesitancy, a significant strength of our research provides precise data on contextual factors of vaccine hesitancy that are specific to the African community in Canada, a racial group that often underrepresented and understudied. Through comprehensive engagement with key partners such as community leaders and community members, the data collected from the study is expected to gain rich insights from centering the voices and perspectives of the participants. Studies have shown that this approach known as Participatory Action Research (PAR) has been used in various settings to understand vaccine hesitancy, such as COVID-19 among specific populations [27–29]. Complementing the results gained from the qualitative component, the regression analysis will aid in identifying the most influential predictors of vaccine hesitancy alongside how these factors may interact with each other. Moreover, when examining the relationship between the independent variables (i.e., factors of vaccine hesitancy) and dependent variable (i.e., vaccine hesitancy), the analysis will measure the strength and direction of the relationship. For example, if the regression model determines that an independent variable such as trust in health systems is a strong predictor of vaccine hesitancy and directionally positive, this would indicate high levels of mistrust is significantly associated with vaccine hesitancy. Therefore, public health initiatives can prioritize this area by creating strategies to facilitate trust in the healthcare system. Similarly, if the analysis determines that a negative relationship exists between an independent variable such as accessing accurate informational resources and vaccine hesitancy as the dependent variable, this can reveal that access to information reduces vaccine hesitancy, suggesting that informational resources can fill knowledge gaps and increase vaccine confidence.

However, a potential limitation of the proposed study is accessibility to the qualitative interviews and quantitative surveys. Individuals who do not have access to technology may be excluded from participating in the study as both components are primarily conducted on Zoom, resulting in possible gaps in our research. Another limitation involves the generalizability of our study. While we are focusing our research on vaccine hesitancy among African communities, we are limiting our reach to African-born individuals present in select Southwestern Ontario cities – London, Windsor and Chatham-Kent which may reduce the applicability of findings for African community members living in different provinces and cities across Canada that were excluded. The cross-sectional survey also presents with limitations in that it provides results that only capture the statistical correlations or associations between the between the independent variables (i.e., drivers of vaccine hesitancy) dependent variable (i.e., vaccine hesitancy). Therefore, we are unable to determine with certainty any cause-and-effect relationships between the variables and whether one factor occurs before another.

By identifying and quantifying the factors influencing vaccine hesitancy in African community members who arrived in Canada, this mix-method study aims to provide culturally specific data that will aid in the creation and improvement of tailored health interventions that can mitigate the burden of vaccine-preventable disease within this population.

## Appendix 1. Survey

*Before beginning the survey, please take a moment to review the terms below*

## Terms

Vaccine: Vaccines are products that help protect people from diseases that spread by stimulating their own immune system

Vaccine Hesitancy: Vaccine hesitancy is when you feel unsure about whether you want to receive a vaccine, even if it is available.

Childhood Vaccines: Vaccines recommended to infants/children from when the child is born until early adolescence. These include:

- DTP (diphtheria, tetanus, and pertussis)
- Polio
- MMR (measles, mumps, rubella)
- Hib (Haemophilus influenzae b)
- Varicella (Chickenpox)
- Rotavirus
- Meningicoccal Meningitis
- Influenza (flu)
- SARS-CoV-2 (Covid-19)

Adolescent Vaccines: During the stage of adolescence, individuals are recommended to receive the following vaccines:

- Human papilloma virus (HPV)
- Meningicoccal Meningitis
- Influenza (Flu)
- Hepatitis B
- SARS-CoV-2 (Covid-19)
- DTP (diptheria, tetanus, and pertussis)

### Demographic questions

**Table:** 

Question	Response Scale
What is your current age?	[Numeric entry]
What is your gender?	Male
	Female
	Non-binary
	Prefer not to answer
	Option not listed (please specify)
What is your country of origin (where were you born)?	List of African countries
What is your religion or spiritual practice?	Buddhism
	Christianity
	Islam
	Hinduism
	Judaism
	Indigenous or Traditional Religion
	No Religion or Spiritual Practice
	Prefer not to answer
Where do you currently reside in Ontario?	London
	Chatham-Kent
	Windsor
What is your level of income	below 10, 000
	10,001–20, 000
	20, 001–40, 000
	40,001–60,000
	60,001–80,000
	80,001–100,000
	100,001 and over
	Prefer not to answer
Including the adults and all the children, how many people live in your household?	[Numeric entry]
What is the highest grade or year of school you have completed?	8th grade or less
	9th-12th grade, no diploma
	High school graduate or GED completed
	Trade, vocational, or business school program
	College Diploma
	Associate degree (AA, AS)
	Bachelor’s degree (BA, BS)
	Master’s (graduate) degree (MS, MA)
	Doctoral degree (PhD)
	Professional degree (MD, MBA, Law)
	Prefer not to answer

### Adult-Focused questions

**Table:** 

Question	Response Scale
Which vaccine(s) have you received?	List
How much do you agree with the following statement?	Do not agree
	Somewhat agree
***If no vaccine doses received***	
If I do not get a COVID-19 vaccine, I will regret it.	Strongly agree
	Very strongly agree
***If any vaccine doses received***	
If I had not gotten a COVID-19 vaccine, I would have regretted it.	
[Do/ Did] you feel the need to get a vaccine so that you [can/ could] do the following? ***Select all that apply.***	Socialize with family
	Socialize with friends
	Attend mass public gatherings (e.g., sporting events or music festivals)
	Travel by plane
	Attend religious services
	Go to a healthcare facility
	Receive medical care
	Go to work or school
	Not wear a mask
	None of the above
How much do you trust the healthcare providers who give you vaccines	Do not trust
	Somewhat trust
	Mostly trust
	Fully trust
How much do you trust the public health agencies that recommend vaccines?	Do not trust
	Somewhat trust
	Mostly trust
	Fully trust
Do you trust that your government is deciding your best interest concerning what vaccines are provided?	Do not trust
	Somewhat trust
	Mostly trust
	Fully trust
Do you believe that vaccine producers are interested in your health?	Do not
	Somewhat
	Mostly
	Fully
Do you trust that vaccines are safe?	Do not
	Somewhat
	Mostly
	Fully
Do you trust that the benefits of vaccines outweigh the side effects?	Do not
	Somewhat
	Mostly
	Fully
If you had to guess, about how many of your family and friends have received the following vaccines:	None
	Some
	Many
	Not sure
Has a doctor, nurse, or other health professional ever recommended that you get a vaccine? If so, which one?	Yes
	COVID-19
	Hep A
	Hep B
	Shingles
	Human papillomavirus (HPV)
	Meningitis
	tetanus
	I have not been recommended
	Not sure
In the last month, have you seen or heard any negative information about the safety or effectiveness of vaccines? If so, which one(s)?	Yes
	COVID-19
	Hep A
	Hep B
	Shingles
	Human papillomavirus (HPV)
	Meningitis
	tetanus
	No
	Not sure
In the past month, how often have you tried to find information about vaccines?	Never
	Rarely
	Sometimes
	Often
	Not sure
[Do/ Did] you feel any of the following tried to influence you to get a vaccine?	Family
	Friends
	Your employer
	Coworkers
	Schools
	Businesses you go to (e.g., restaurants or grocery stores)
How much do you agree with the following statement? I have a responsibility to get vaccinated to protect others.	Do not agree
	Somewhat agree
	Strongly agree
	Very strongly agree
How difficult [would it be for you/ was it for you] to get a vaccine?	Not at all difficult
	A little difficult
	Somewhat difficult
	Very difficult
Have you heard of cash prizes or other rewards being offered in your area to people who get a vaccine?	Yes
	No
	Not sure
Does your work or school require you to get a vaccine?	Yes
	No
	Unemployed/Not applicable (not in school, home schooled)
	Not sure
How much do you agree with the following statement: I can get a vaccine if I want to.	Do not agree
	Somewhat agree
	Strongly agree
	Very strongly agree
Many things might make it difficult to get a vaccine. Which of the things in this list [made]/[makes] it difficult for you?	Getting an appointment online
	Not knowing where to get vaccinated
	Hard to get to vaccination sites
	Vaccination sites aren’t open at convenient times
	None of these
How does offering cash prizes or other rewards to get a vaccine affect your trust in a vaccine?	Decreases my trust
	Has no influence on my trust
	Increases my trust

### Child-focused questions

**Table:** 

Do you have any children? If yes, how many?	Yes
	No
	1
	2
	3
	4
	5
	More than 5
What age is your child/children? (How old)	Child 1 (first born)
	Child 2 (second-born)
	Child 3 (third-born)
	Child 4 (fourth-born)
	Child 5 (fifth born)
	Other children
What is your child’s/children’s gender?	*Respondents will have the ability to select one of the options below for each child*
	Male
	Female
	Non-binary
	Prefer not to answer
	Option not listed (please specify)
How much do you agree with the following statement: “If I do not get [child’s name] a vaccine, I will regret it.”	*Respondents will have the ability to select one of the options below for each child*
	Do not agree
	Somewhat agree
	Strongly agree
	Very strongly agree
	Don’t know
	Refused
[Do/ Did] you feel the need to get your child/children a vaccine so that they could do the following? Check all that apply.	Socialize with family
	Socialize with friends
	Attend mass public gatherings (e.g., sporting events or music festivals)
	Travel by plane
	Attend religious services
	Go to a healthcare facility
	Receive medical care
	Go to work or school
	Not wear a mask None of the above
How concerned are you about your child/children getting sick because of an infectious disease?	*Respondents will have the ability to select one of the options below for each child*
	Not at all concerned
	A little concerned
	Somewhat concerned
	Very concerned
	Don’t know
	Refused
Once your child/children is eligible for a vaccine, how important do you think getting a vaccine is to protect [child’s name] against infectious diseases?	*Respondents will have the ability to select one of the options below for each child*
	Not at all important
	A little important
	Somewhat important
	Very important
	Don’t know
	Refused
How safe do you think vaccines are for your child/children?	*Respondents will have the ability to select one of the options below for each child*
	Not at all safe
	Somewhat safe
	Very safe
	Completely safe
	Don’t know
	Refused
To your knowledge, which vaccines has your child/children received?	*Respondents will have the ability to select one of the options below for each child*
	DTP (Diphtheria tetanus toxoid and pertussis)
	Polio
	MMR (measles, mumps, rubella)
	Hib (Haemophilus influenzae b)
	Varicella (Chickenpox
	Rotavirus
	Meningicoccal Meningitis
	Influenza (flu)
	Human papillomavirus (HPV)
	COVID-19
Does your child’s/children’s school require them to get a vaccine to attend in-person classes?	Yes
	No
	Not in school, home schooled
	Not know
	Refused
How much do you agree with the following statement: I can get my child/children a vaccine if I want to.	Do not agree
	Somewhat agree
	Strongly agree
	Very strongly agree
Many things might make it difficult to get a vaccine for your child. Select which of the following makes it difficult for your child/children.	Getting an appointment online
	Not knowing where to get vaccinated
	Hard to get to vaccination sites
	Vaccination sites aren’t open at convenient times
	None of these
Does your child/children have a health condition that may put them at a higher risk for infection?	Yes
	No
	Not sure
	Prefer not to answer
If you answered yes to the question above, can you specify what the health condition is?	Please list below
	Prefer not to answer
